# Engineered IgG1-Fc Molecules Define Valency Control of Cell Surface Fcγ Receptor Inhibition and Activation in Endosomes

**DOI:** 10.3389/fimmu.2020.617767

**Published:** 2021-02-15

**Authors:** Elizabeth M. Bailey, Amit Choudhury, Harika Vuppula, Daniel F. Ortiz, John Schaeck, Anthony M. Manning, Carlos J. Bosques, Adam D. Hoppe

**Affiliations:** ^1^Department of Chemistry and Biochemistry, South Dakota State University, Brookings, SD, United States; ^2^BioSystems Networks and Translational Research, South Dakota State University, Brookings, SD, United States; ^3^Momenta Pharmaceuticals, Cambridge, MA, United States

**Keywords:** macrophage, Fcγ receptor, antibodies, immune complex, autoimmunity, monocyte, inhibitor, single molecule

## Abstract

The inhibition of Fcγ receptors (FcγR) is an attractive strategy for treating diseases driven by IgG immune complexes (IC). Previously, we demonstrated that an engineered tri-valent arrangement of IgG1 Fc domains (SIF1) potently inhibited FcγR activation by IC, whereas a penta-valent Fc molecule (PentX) activated FcγR, potentially mimicking ICs and leading to Syk phosphorylation. Thus, a precise balance exists between the number of engaged FcγRs for inhibition versus activation. Here, we demonstrate that Fc valency differentially controls FcγR activation and inhibition within distinct subcellular compartments. Large Fc multimer clusters consisting of 5-50 Fc domains predominately recruited Syk-mScarlet to patches on the plasma membrane, whereas PentX exclusively recruited Syk-mScarlet to endosomes in human monocytic cell line (THP-1 cells). In contrast, SIF1, similar to monomeric Fc, spent longer periods docked to FcγRs on the plasma membrane and did not accumulate and recruit Syk-mScarlet within large endosomes. Single particle tracking (SPT) of fluorescent engineered Fc molecules and Syk-mScarlet at the plasma membrane imaged by total internal reflection fluorescence microscopy (SPT-TIRF), revealed that Syk-mScarlet sampled the plasma membrane was not recruited to FcγR docked with any of the engineered Fc molecules at the plasma membrane. Furthermore, the motions of FcγRs docked with recombinant Fc (rFc), SIF1 or PentX, displayed similar motions with D ~ 0.15 μm^2^/s, indicating that SIF1 and PentX did not induce reorganization or microclustering of FcγRs beyond the ligating valency. Multicolor SPT-TIRF and brightness analysis of docked rFc, SIF1 and PentX also indicated that FcγRs were not pre-assembled into clusters. Taken together, activation on the plasma membrane requires assembly of more than 5 FcγRs. Unlike rFc or SIF1, PentX accumulated Syk-mScarlet on endosomes indicating that the threshold for FcγR activation on endosomes is lower than on the plasma membrane. We conclude that the inhibitory effects of SIF1 are mediated by stabilizing a ligated and inactive FcγR on the plasma membrane. Thus, FcγR inhibition can be achieved by low valency ligation with SIF1 that behaves similarly to FcγR docked with monomeric IgG.

## Introduction

Immune complexes (IC) containing immunoglobulin G (IgG) are a hallmark of many autoimmune diseases and inflammatory reactions. The activation of Fcγ receptors (FcγR) on macrophages by IC-IgG contributes to cytokine signaling and inflammatory pathogenesis in autoimmunity including the recruitment of neutrophils, monocytes, T cells, natural killer cells (NK cells), and additional macrophages (1, 2). In systemic lupus erythematosus (SLE), a chronic autoimmune disease, IC-IgG deposits in organs and tissues (3) and are recognized by FcγRs on macrophages, monocytes and neutrophils (4). In SLE, macrophages are overwhelmed and unable to clear the ICs leading to continued macrophage activation, sustained secretion of pro-inflammatory cytokines, autoantibody production, tissue damage and acute phases of the disease (5). Macrophages also present antigen to T cells, which promotes their activation and additional tissue damage (5). Thus, inhibiting FcγR activation and disrupting this inflammatory circuit is an attractive therapeutic strategy for treating autoimmunity (6).

Immune reactions to IC-IgGs are governed by activating and inhibitory FcγRs. Activating FcγRs (FcγRI, FcγRIIa, FcγRIIc, and FcγRIIIa/b) signal *via* immunoreceptor tyrosine-based activation motifs (ITAMs) found in the cytoplasmic tail of FcγRIIa/c or within the ‘common gamma chain’ (FcRγ) associated with FcγRI and FcγRIIIa/b. FcγRIIb contains an immunoreceptor tyrosine-based inhibition motif (ITIM). IgG bound to ICs or pathogen surfaces bind FcγRs leading to clustering and phosphorylation of ITAM phosphotyrosines by the Src family kinases, including Lyn. Recruitment of Syk kinase to phosphorylated ITAM is the hallmark of FcγR activation (2, 7, 8), where it promotes phosphorylation of additional signaling molecules including phosphotidylinositiol-3-kinase and Bruton’s tyrosine kinase (BTK) leading to signal amplification (9, 10). Phosphorylation of the ITIM within FcγRIIb allows docking of SHIP-1, a 5’-phophoinosital phosphatase that negatively regulates inflammatory responses triggered by ICs (2). Downstream effects of FcγR activation include antibody-dependent cell-mediated cytotoxicity (ADCC), and phagocytosis (ADCP) (1, 2, 11), as well as cytokine production promoting inflammation and autoimmunity in NFAT/NFkb dependent pathways (2).

FcγR inhibition is a therapeutic target for autoimmune diseases with demonstrated potential for treating IC-associated autoimmune diseases (6, 12–15). Monomeric IgG Fc fragments and IVIG are effective in treating rheumatoid arthritis models in mice and humans for various autoimmune diseases such as Kawasaki disease and acute immune thrombocytopenic purpura (ITP) (13–15). While this strategy can provide therapeutic benefit, it requires large doses as the Fc-FcγR binding is 1:1 and may be displaced by polyvalent ICs. An alternative strategy currently in clinical trial, is to create multivalent Fc molecules that have high affinity for the FcγR, but do not activate it, thereby preventing the IC-IgG binding and providing long-lived inhibition (6). Disruption of FcγR signaling in turn inhibits pro-inflammatory response and alleviates inflammation and tissue damage and limits B and T cell activation and autoantibody production (2, 5, 6). Previously, we demonstrated that the valency of engineered Fc molecules could potently inhibit or potently activate FcγRs (6). Specifically, pentameric Fc arranged in an X-geometry (PentX), was a strong agonist for FcγRs, mediating potent Syk phosphorylation, whereas the trimer, SIF1, inhibited FcγR activation by IC-IgG both *in vitro* as well as in animal autoimmune models (6). How these two constructs effect differential outcomes for FcγR signaling offers new mechanistic insights.

Understanding the biophysical mechanisms of FcγR activation is critical for elucidating robust strategies using engineered Fc molecules as inhibitors. In the context of phagocytosis, engagement of FcγR with hundreds to thousands of surface-associated IgG promotes FcγR clustering on the micrometer length scale driven by the macrophage cytoskeleton (16). For IC-IgG, recent superresolution experiments on fixed cells suggest that FcγRs exist as both monomers and in pre-formed clusters confined by the cytoskeleton and that IC-IgG prompts clustering of additional FcγRs (17, 18). Furthermore, IC-IgG immobilization of FcγR clusters is thought to promote internalization and degradation of the ICs in the lysosome (19), IC-mediated signaling from endosomes along the endocytic pathway have not been evaluated. Here, we defined the effect of Fcs with defined valency on the movements of FcγRs on the cell surface, endocytosis and Syk recruitment to the plasma membrane and FcγR bearing endosomes. Our findings shed new light on FcγR inhibition at the plasma membrane and illustrate that endocytosis may lower thresholds for FcγR activation.

## Methods

### Cell Culture

THP-1 cells were cultured in RPMI-1640 Medium (ATCC 30-2001) and maintained at 100,000–200,000 cells per milliliter. Differentiation of THP-1 cells into macrophage-like cells with high levels of FcγR was achieved in a 48-h incubation in RPMI containing 50 ng/ml Phorbol 12-myristate 13-acetate, 97% (PMA, Acros Organics, AC356150010) and rested for 24-h in RPMI without PMA. Lentiviral vectors were produced in 293T cells by transfection with Syk-mScarlet-pLJM1 (https://benchling.com/s/seq-VHzJFZG9UJ4vW2bJ2Rmp/edit), pPAX2 (Addgene plasmid # 12260), pVSVG (Addgene plasmid #8454), using polyethylenimine (PEI). After 48 h, lentivirus was harvested, centrifuged, and added to THP-1 cells. After the 48-h transduction, cells were selected with Blastidin (10 μg/ml) for 2 days.

PMA differentiated THP-1 cells were characterized using fluorescently conjugated Fab fragments (to exclude Fc-mediated binding to FcγRs) of anti-hCD64 (Santa Cruz Biotechnology sc1184) and anti-hCD32 (StemCell Technologies 60012). Fab fragments were generated using 62.5 μg of each antibody using the Pierce Fab micro preparation kit (Thermo Fisher 44685). The anti-CD64 and anti-CD32 Fab fragments were labeled with AF647 NHS ester and used to label THP-1 and differentiated THP-1 cells. Cells were blocked with dPBS+1% FBS for 15 min on ice before adding the Fab fragments diluted into dPBS with 1% FBS at a 1:8 dilution of 1.14 μM labeled Fab fragments (final concentration of approximately 0.18 μM), incubated 20 min on ice, spun down at 800 g for 3 min and resuspended in cold Hank’s Balanced Salt Solution (HBSS, Corning™21023CV) and kept on ice until analyzed by the BD Accuri C6 flow cytometer (**Supplementary Figure 1**). We were unsuccessful in preparing anti-CD16 Fab fragments.

Differentiated THP-1 were evaluated for their phagocytic activity by dropping a 30:1 MOI of biotinylated and anti-IgG2a opsonized sRBCs (sheep red blood cells) on differentiated THP-1 cells. Phase contrast microscopy using an inverted fluorescent microscope was used for a qualitative analysis to observe phagocytosed sRBCs. PMA differentiated THP-1 cells had an elevated phagocytic capacity (data not shown).

Fetal liver macrophages expressing Cas9 (FLM^Cas9^) cell cultures were generated from gestational day 15–19 mouse fetuses from B6J.129(Cg)-Igs2tm1.1(CAG-cas9*)Mmw/J mice (The Jackson Laboratory, Stock No. 028239, Bar Harbor, ME) (20) in accordance with South Dakota State University Institutional Animal Use and Care Committee. Liver tissue was mechanically dissociated using sterile fine-pointed forceps and a single-cell suspension was created by passing the tissue through a 1 ml pipette tip. Cells were plated on non-tissue culture treated dishes and kept in growth and differentiation medium containing the following: 20% heat-inactivated fetal bovine serum; 30% L-cell supernatant, a source of MCSF and 50% Dulbecco’s modified growth medium containing 4.5 g/L glucose, 110 mg/L sodium pyruvate, 584 mg/L-glutamine, 1 IU/ml penicillin and 100 μg/ml streptomycin. FLM were cultured for at least 8 weeks prior to transduction and experiments.

Gene disruption of Syk in FLM^Cas9^ cells was conducted by lentiviral integration of an sgRNA and puromycin drug selection marker following the approach outlined above in 293T cells with the transfer plasmid replaced by plentiGuide-Puro (Addgene plasmid #52963) targeting an early exon (sgRNA targeting: ATTGCACTACCGCATTGACA). 293T supernatant was transferred onto the FLM^Cas9^ cells and puromycin selection of 1 µg/ml was carried out for 48h.

### Multimeric Fc Molecules

All Fc molecules were prepared and labeled at Momenta Pharmaceutics using the methods described in (6). Size exclusion chromatography was used to validate the purity of the labeled molecules. **Supplementary Figure 1** provides the SEC data demonstrating that all molecules were of high purity and of the expected size.

### Live Cell Imaging

For imaging cells on glass, cells were differentiated directly on ethanol flamed 25 mm coverslips (Number 1.5, Thermo Fisher) or onto 96-well glass bottom plates (Dot Scientific, MGB096-1-2-LG-L) for high content experiments. The 25 mm coverslips were imaged in AttoFluor chambers (Thermo Fisher). TIRF-based imaging which was conducted with an inverted microscope built around a Till iMIC (Till Photonics, Germany) equipped with a 60 × 1.49 N.A. oil immersion objective lens, enclosed in a custom environmental chamber to keep the samples at 35–37°C. The entire microscope setup and centering of the back focal plane was previously described in (21). Excitation for TIRF was provided by either a 561 nm laser, for DL594 labeled molecules or Syk-mScarlet, or a 488 nm laser for the AF488 labeled molecules. Single point TIRF was used for fast imaging of single particles while TIRF 360 was used to create uniform TIRF illumination for moderate speed particle tracking by steering the laser at the back-focal plane (22). The microscope was custom-built based on iMIC system (TILL Photonics, Munich, Germany) with 60x 1.49 oil immersion objective lens (Olympus, Tokyo, Japan).

For confocal experiments on undifferentiated THP-1 cells, cells were plated on 25mm coverslips that had been coated with 0.01% Poly-L-Lysine solution (Sigma, A-005-M) and imaging was performed on a TIL photonics Andromeda Spinning Disk Confocal, using a 60x oil 1.4 oil immersion objective lens. Cells were plated overnight and incubated with 100 µg/ml of either the FcM AF488 or the PentX AF488 for 5 min before imaging. As in the TIRF imaging, cells were maintained at 35–37°C in HBSS.

High content microscopy of FLM cell were plated into 96-well glass bottom plates (Dot Scientific, MGB096-1-2-LG-L) 2–4 h prior to imaging. Images were captured on an ImageXpress Micro XLS (Molecular Devices, Sunnyvale, CA) equipped with a 40 × 0.90 N.A. objective lens. Macrophages were marked with HCS NuclearMask™ Blue Stain (ThermoFisher scientific) for identification and AF647 or DL647 fluorescence was collected using integrated filter cubes for far red and red respectively. For antibody dependent phagocytosis, sheep red blood cells (sRBC) were biotinlayted using a mixture of NHS-biotin and NHS-AF647 (Thermo Fisher scientific) and opsonized with murine IgG2a anti-biotin [3E6] (ab36406, Abcam). IgG-sRBCs were added at an MOI of 20 and incubated for 40 min prior to imaging.

### Supported Lipid Bilayers

Supported lipid bilayers (SLB) were formed by spontaneous fusion of lipid vesicles. For the bilayers, small unilamellar vesicles were prepared by mixing DSPE-PEG(2000)-DBCO (Avanti Polar Lipids 880229) and POPC (850457 Avanti Polar Lipids) at a molar ratio of 1:1,000 with total lipid concentration of 500 μM (380 µg/ml), in chloroform and dried using vacuum centrifugation. The lipid film was resuspended in 1 milliliter PBS (without Ca^+^ and Mg^+^) by vortexing and then sonicating for 5 min using a bath sonicator, followed by extrusion (Avanti Polar Lipids 610000) through 100 nm filter at least 13 times (Whatman Nucleopore Track-Etch 100 nm membrane). The 500 uM stock of lipids were diluted 1:6 in 2 mM Mg^2+^ PBS. The bilayer was formed by pipetting the diluted lipids onto 25 mm Piranha acid [H_2_SO_4_ (30%, *v/v*):H_2_O_2_ (3:1, *v/v*)] cleaned coverslip (held in Leiden chambers) and incubated at 37°C for 20 min. Following incubation, bilayers were then submerged in water at 42°C to remove excess liposomes. The water was exchanged with PBS followed by HBSS for imaging.

The cRGD {Cyclo[Arg-Gly-Asp-D-Phe-Lys(Azide)]} was linked to liposomes *via* a copper free click chemistry reaction following resuspension of the dried lipids. Here, 1 μg cRGD-Azide (Peptides International RGD-3749-PI) was reacted to the 380 μg of liposomes, so that there was an excess (about 3 fold) of cRGD-Azide to liposomes. The cRGD-Azide was added to the liposome solution prior to vortexing. The lipids were then sonicated and extruded (to form the liposomes 100 nm in size as described above.

IgG-opsonized bilayers were prepared as described in but were instead comprised of DSPE-PEG(2000) Biotin (Avanti Polar Lipids 880129) mixed with POPC (850457 Avanti Polar Lipids) at a molar ratio of 1:1000 with total lipid concentration of 500 μM. Alexa Fluor 647 NHS ester (Thermo Fisher Invitrogen) was conjugated to anti-Biotin IgG (3E6) (Abcam ab36406) for antibody fluorescent labeling. The labeled antibody was incubated with the 0.1% PEG-biotin SLB at 37°C for 30 min in dPBS. Excess IgG was washed with HBSS.

### Fc Inhibitor Treatments

For single particle assays on the cRGD bilayer, differentiated THP-1 cells were removed from the plastic dish using cold dPBS and then exposed to either 0.03 μg/ml rFc-DL594, 0.11 μg/ml SIF1-DL594, or 0.17 μg/ml PentX-DL594 (~0.7 nM each) in suspension at 4**°**C for 30 min . Cells were washed and resuspended in HBSS and dropped on the supported lipid bilayers at 37°C. For high dosage treatments on glass, Fc multimers labeled with AF488 were held at the same mass, 33 μg/ml, 66 μg/ml, or 100–110 μg/ml doses for the particle tracking data. These were treated at 37°C for 5 min and then washed with HBSS and imaged at times indicated. For brightness analysis, Fc multimers were all 0.4 μM for each to keep the total number of molecules the same for each.

For FcM-AF488 and PentX-AF488 treatment of undifferentiated THP-1 cells, plated on Poly-L-Lysine coated coverslips, 100 µg/ml for each was incubated for 5 min prior to imaging. Due to lack of cell adherence, cells were not washed, so cells were continuously exposed over the duration of imaging.

### Image Processing

For fiducial data collection and image registration, single images were registered using calibration images acquired simultaneously on each of the four EMCCD detectors. A single image of 200 nm green beads (Life Technologies, Carlsbad, CA) immobilized on a glass coverslip were excited using 445 nm excitation. Coordinates for registration were determined using the MATLAB (The MathWorks, Inc., Natick MA) cpselect tool. A rigid affine transformation was used to transform all points onto the red channel. Image montages were generated using ImageJ, and due to unintended photobleaching in some data sets, the Bleach Correction tool was used.

U-track (Danuser Lab) was used for single particle tracking using the Gaussian detection (23). Motion of the Fc multimer tracks were classified by the divide-and-conquer moment scaling spectrum (DC-MSS) method (24). Syk detections per µm^2^ per second were measured using Gaussian detection in U-track from a cropped region that excluded the cell edge to prevent anomalous detections from the transition between cell background and cell-free background regions. For the brightness analysis, point source detection in U-track was carried out from single frames.

## Results

### Engagement of FcγRs by Large Complexes of Fc Recruit Syk to the Plasma Membrane, Whereas PentX Recruits Syk to Endosomes

**Figure 1:**
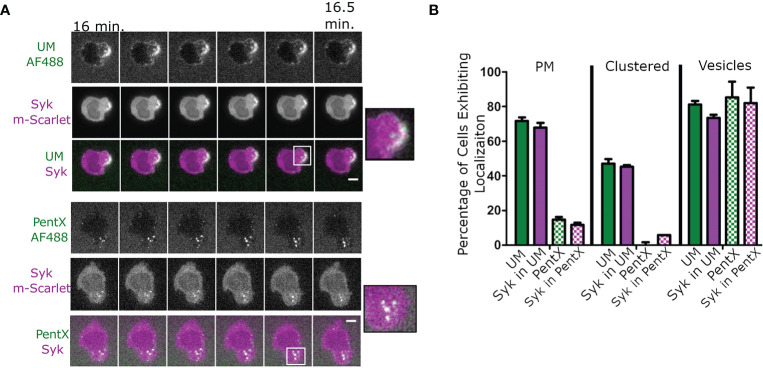
PentX recruits Syk to endosomes, whereas multimeric Fc-FcγR complexes recruit Syk to the plasma membrane (PM). **(A)** Confocal imaging of Syk-mScarlet localization in THP-1 cells relative to fluorescently labeled PentX-AF488 or unconstrained Fc multimer (UM-AF488). By 15 min of incubation with Fc constructs, Syk-mScarlet localized to PM patches of UM-AF488 and occasionally on endosomes, whereas Syk-mScarlet localized only to endosomes PentX-AF488 (scale bar, 5µm). **(B)** The percentage of cells exhibiting localization of UM-AF488 or PentX-AF488 or Syk-mScarlet to PM, PM-clusters or intracellular vesicles following15 min of incubation (error bars are standard deviation, n= 34 and 53 cells for PentX and UM respectively, two independent experiments).

To determine the location of FcγR activation in response to IC-IgG, fluorescently tagged polymeric multimers of Fc (uncontrolled multimer, UM (6), comprised predominately of 10–40 Fc domains similar to IC-IgG were added to THP-1 cells. Syk-mScarlet expressing THP-1 cells formed patches of UM clusters at the cell surface that recruited Syk-mScarlet within 30 min (**Figure 1A**, **Supplementary Video 1**). Intracellular vesicles bearing UM and Syk-mScarlet were also observed **(Figure 1A**). This result was consistent with the conventional model in which clustering of FcγRs by binding IC-IgG or FcϵRI binding IC-IgE, leads to phosphorylation of ITAMs, Syk recruitment and subsequent internalization (19, 25). We anticipated that PentX, which potently promoted Syk phosphorylation (6), would behave similarly. Surprisingly, PentX did not produce clusters at the plasma membrane (PM). Rather, PentX bound to the cell surface and then within 15 min localized to small and large intracellular vesicles, where it recruited Syk-mScarlet (**Figure 1A**, **Supplementary Video 2**). Quantification of these confocal images indicated that Syk-mScarlet was weakly or not recruited to the PM of cells exposed to PentX, whereas UM readily accumulated Syk-mScarlet at the PM (**Figure 1B**). Thus, PentX did not significantly activate FcγRs on the cell surface, but rather selectively promoted FcγR activation and accumulation in endosomes. These results reveal that Fc valency controls the subcellular distribution and activation of FcγR.

### SIF1 and rFc Linger on the Plasma Membrane and Do Not Recruit Syk

**Figure 2:**
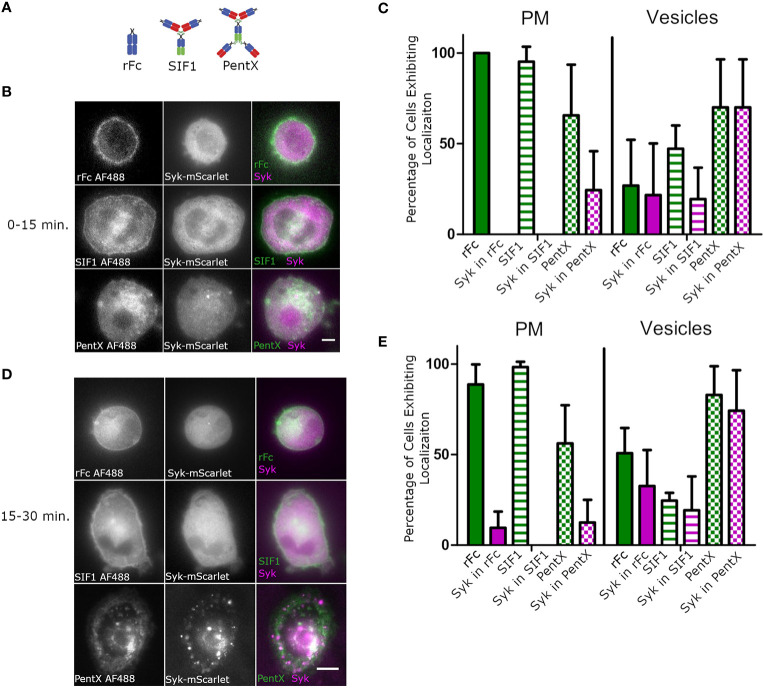
SIF1 and rFc remain on PM and do not recruit Syk. **(A)** Molecular structure cartoons from (6). **(B)** Representative Hi-Lo images of Fc-AF488 constructs (100 µg/ml) and Syk-mScarlet distribution within 15 min. of exposure of differentiated THP-1 cells to Fc constructs (scale bar, 5µm). **(C)** Quantification of Fc-AF488 molecules and Syk-mScarlet localization within 15 min. post treatment (error bars = standard deviation, n=21 cells rFc, 16 cells SIF1, 14 cells across 3 experiments). Cells were categorized as exhibiting Fc and Syk localization at either the plasma membrane or intracellular, typically within distinct vesicles. Cells could show both localizations. **(D)** Representative Hi-Lo images of Fc-AF488 constructs (100 µg/ml) and Syk-mScarlet distribution between 16 min. and 30 min. of Fc treatment of differentiated THP-1 cells (scale bar, 5µm). **(E)** Data taken on cells within 16–30 min of Fc treatment were quantified in the same way as panel **(C)** (error bars = standard deviation, n=35 cells rFc, 35 cells SIF1, 33 cells across three experiments).

We next sought to compare the effects of monomeric, trimeric and pentameric Fc-molecules (**Figure 2A**) on the activation of FcγRs on subcellular membranes. Using HiLo microscopy, which allowed greater sensitivity than confocal microscopy, but with similar optical sectioning (26), we imaged the distributions of monomeric rFc, the inhibitory Fc trimer (SIF1), and PentX relative to Syk-mScarlet in THP-1 cells differentiated toward macrophages by 48 h exposure to PMA which increased expression of FcγR1 and adherence to glass coverslips (**Supplementary Figure 2**). Since PentX was internalized by ~15 min, the data were divided between early timepoints, below 15 min (**Figures 2B, C**), and later timepoints, 15–30 min (**Figures 2D, E**). In contrast to PentX which accumulated on endosomes, rFc and SIF1 were lingered on the PM, with a small fraction trafficking on small endosomes for at least 30 min and neither recruited Syk-mScarlet to the PM (**Figures 2B–E**) consistent with (6). The high sensitivity of the HiLo approach revealed many larger PentX containing endosomes that extensively recruited Syk-mScarlet with some variability between endosomes (**Figures 2B–E**). A basal level of endocytosis appeared to internalize rFc and SIF1 over time, but Syk-mScarlet did not colocalize with rFc or SIF1 in vesicles. These observations are consistent with the known inhibitory activity of SIF1 and indicate that it traps FcγR in a state mimicking monovalent Fc binding, whereas PentX either promotes FcγR internalization or prevents recycling of FcγRs to the PM by their activation on endosomes.

### rFc, SIF1 and PentX Do Not Recruit Syk to the PM Unlike Surface Associated IgG During Frustrated Phagocytosis

**Figure 3:**
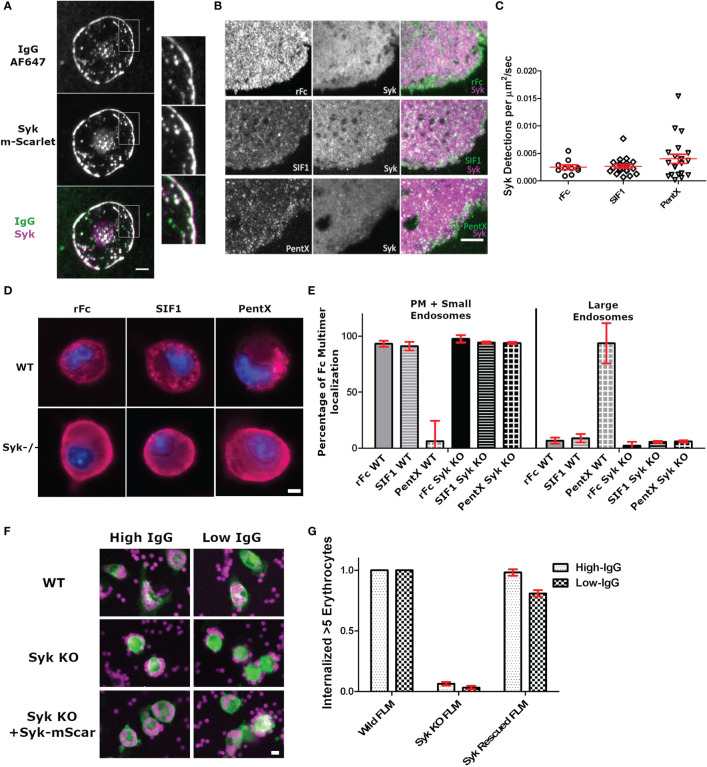
Syk is recruited to surface associated IgG bound FcγR, but not those docked with low Fc valencies. **(A)** TIRF imaging of intense Syk-mScarlet recruitment during frustrated phagocytosis by a THP-1 cell of SLB presenting AF647-IgG. Note that too many Syk-mScarlet molecules are recruited for single molecule detection. Image is representative of 20+ cells. Scale bar is 5µm. **(B)** Single particle detections of Syk-mScarlet molecules on the surface of cells docked with AF488-Fc molecules (100 µg/ml rFc, SIF1, or PentX). Scale bar is 5µm. **(C)** The frequency of Syk-mScarlet detections observed. No statistical significant difference. Data represents 10 rFc-treated cells, 20 SIF1-treated cells, and 20 PentX-treated cells from a single day that represents results of two experiments. Bars are mean +/- standard error of the mean. **(D)** Wild-type and Syk-KO FLMs exposed to DL594-rFc, SIF1 or PentX. Scale bar is 5µm. **(E)** Quantification of localization frequencies of DL594-rFc, SIF1, or PentX in WT and Syk-KO FLMs. ~150 cells/condition from two independent experiments. Error bars are standard deviation. **(F)** Antibody dependent phagocytosis of IgG-sRBC labeled with AF647 by WT, Syk-KO, and Syk-KO+Syk-mScarlet expressing FLMs. Scale bar is 10µm. **(G)** Quantification of the frequency of macrophages internalizing 5 or more IgG-sRBC. ~120 cells/condition. Error bars are standard deviation.

Given that PentX recruited Syk to endosomes, we sought to determine if Syk was initially recruited to FcγRs at the PM and if there was any reversible recruitment of Syk to rFc or SIF1-engaged FcγRs. Total internal reflection fluorescence (TIRF) microscopy provides an exquisite view of single molecule movements at the PM while eliminating contaminating fluorescence from other regions of the cell (16). Differentiated THP-1 cells robustly engaged AF647-IgG docked on supported lipid bilayers (SLB) by spreading and recruiting many hundreds to thousands of Syk-mScarlet molecules per µm^2^ to the advancing lamellipodia within ~–1–5 min of contacting the surface (**Figure 3A**, **Supplementary Video 3**). Notably, the patches of AF647-IgG, to which Syk-mScarlet was recruited, were reminiscent in structure to those created by UM molecules **(Figure 2**, compare with **Figure 1A**), suggesting the binding of surface associated IgG or high-valency IgG molecules such as UM, creates large patches of FcγRs on the PM to which Syk is recruited. Conversely, TIRF imaging of the adherent plasma membrane of Syk-mScarlet expressing THP-1 docked with saturating concentrations (100 µg/ml) of labeled AF488-rFc, SIF1 or PentX revealed a predominately uniform dim fluorescence emanating from Syk-mScarlet molecules diffusing in the cytoplasm. Syk-mScarlet molecules transiently sampled the plasma membrane, as seen by localized bursts of fluorescence, however, the generally did not frequently colocalize with AF488-rFc, SIF1 or PentX docked to FcγRs **(Figure 3B** and **Supplementary Videos 4**–**6**). We quantified the number of Syk localizations to the plasma membrane, since their duration was too short to reliably track, and found only a slight, non-statistically significant trend toward more localizations for PentX and indistinguishable frequency for SIF1 and rFc (**Figure 3C**). Most of these localizations were fleeting, lasting around 1 s or less. Thus, unlike surface associated IgG, the soluble, multimeric molecules do not significantly recruit Syk to the PM.

### Syk Contributes to the Endocytic Traffic of PentX

To determine if Syk was required for the accumulation of PentX in endosomes, we created murine fetal liver macrophages (FLMs) from Cas9 mice and knocked out Syk using lentiviral expression and selection for single guide RNA (sgRNA) targeting an early exon to produce Syk^sgRNA^ FLMs. Similar to differentiated THP-1 cells, DL594-rFc and DL594-SIF1 lingered on the PM and were small endosomes, whereas DL594-PentX was internalized and trafficked to large endosomes following 20 min of incubation **(Figure 3D**). Notably, more small endosomes containing DL594-rFc or DL594-SIF1 were observed than in THP-1 cells, potentially due to the elevated endocytic and macropinocytic activity of these cells relative to THP-1 cells (data not shown). Quantification by high content microscopy indicated that the failure of Syk^sgRNA^ FLMs to traffic DL594-PentX into large endosomes at 20 min post exposure (**Figures 3D, E**). Additionally, small vesicles were observed below the plasma membrane for DL594-rFc, DL594-SIF1 and DL594-PentX suggesting that Syk controls the endocytic traffic of the activating PentX and potentially recycling to the cell surface. To confirm the knockout phenotype of the Syk^sgRNA^ FLMs and functionality of the Syk-mScarlet probe we performed high content microscopy of WT, Syk-KO and Syk-KO/Syk-mScarlet rescued cells. WT FLMs robustly internalized IgG-opsonized sheep red blood cells (IgG-sRBC) whereas Syk-KO cells were incapable of engulfing sRBCs (**Figures 3F**, **G**). Expression of the Syk-mScarlet, followed by drug selection, showed a striking rescue of sRBC engulfment in Syk^sgRNA^ FLMs indicating that the Syk-mScarlet construct should not interfere with the function of the endogenous Syk molecules in the THP-1 cells and should provide a functionally accurate view of Syk dynamics. We interpret these results to indicate that at the PM low valency (<5) Fcs may do not significantly recruit Syk or maintain it actively docked at the PM and that once internalized on endosomes. However, complexes of 5 FcγRs can robustly recruit Syk to endosomes where signaling unique from that encountered at the plasma membrane may occur (**Figure 2**).

### rFc, SIF1, and PentX Display Similar Motions at the Plasma Membrane

**Figure 4:**
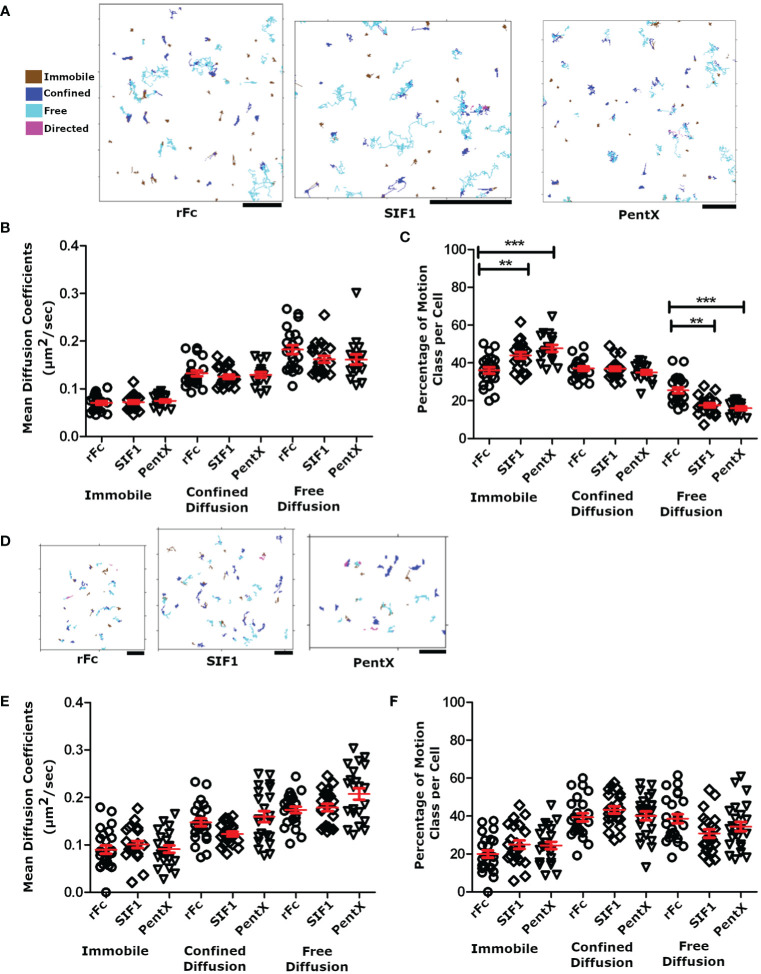
Motion analysis of Fc molecules docked to FcγR. **(A)** SPT tracks following analysis with DC-MSS, from TIRF imaging of THP-1 cells treated with 100 μg/ml rFc, SIF1 or Pentx, color coded by motion class. Note that transitions in track classifications (e.g. confined to free) can be observed within single tracks. All scale bars represent 2µm. **(B)** The average diffusion coefficient for each cell by diffusion class. **(C)** The percentage of tracks for each diffusion class. Data in panels **A–C** are from 22 rFc treated cell, 21 SIF1 treated cells, and 17 PentX treated cells from a single day experiment but are representative of multiple replicates. **(D)** Representative SPT tracks of THP-1 cells treated with fluorescently labeled Fc molecules and attached to supported lipid bilayers displaying cycloRGD, at sub-activating/inhibiting doses of 0.7 nM (0.03 µg/ml rFc, 0.11 µg/ml SIF1, and 0.17 µg/ml PentX). All scale bars represent 2µm. **(E)** The average diffusion coefficient for each cell plotted by diffusion class at 0.7 nM. **(F)** The percentage of tracks for each diffusion at 0.7nM. Data from panels **(D–F)** are from 24 rFc treated cells, 22 SIF1 treated cells, and 22 PentX treated cells taken across three different experiments. Red bars are the mean +/- standard error of the mean. Significance was calculated using Tukey’s one-way ANOVA where (*P < 0.05, **P < 0.005, *** < 0.0005).

Since Syk was not recruited to low valency Fc-FcγR complexes on the plasma membrane, we speculated that the SIF1- and PentX-FcγR complexes were not forming clusters and have similar diffusive motions on the PM. Conversely, decreased mobility of these complexes would be an indicator of FcγR clustering or the association with endosomal structures. To test this idea, THP-1 cells were treated with 100 µg/ml of DL594-Fc molecules at fully activating (PentX) or inhibiting (SIF1) concentrations (6) and plated onto coverslips. The adherent surface was imaged by TIRF microscopy at 18 frames per second for 200 frames. SPT trajectories from these movies were found using U-track (23) and classified by the divide-and-conquer moment scaling spectrum (DC-MSS) method (24) and color coded by the diffusion type as freely diffusing, confined diffusion and immobile (**Figure 4A**). Here ‘immobile’ from the DC-MSS analysis may reflect highly confined trajectories at the noise/localization limit for the organic fluorophores used, and thus a diffusion coefficient is reported for this class, even though it likely reflects an upper bound. Overall, the quality of tracking was quite high indicating that the TIRF microscopy approach provided a sufficiently low background for imaging an organic fluorophore (DL594) rather than the more frequently used quantum dots. The motions of the Fc constructs displayed similar proportions of each motion class and could be observed transitioning between classes (frequently between confined and free diffusion, dark blue to or from light blue, **Figure 3A**). These motion types are consistent with Fc-FcγR complexes interacting with actin corrals, lipid rafts, and protein islands (27–30). As predicted by the lack of Syk recruitment, only small differences in diffusion constants and types of motions were observed across rFc, SIF1 and PentX (**Figure 4B**). A small increase in the fraction of immobile complexes was observed for PentX and SIF1 over rFc along with a concomitant reduction in freely diffusing complexes (**Figure 4C**). We interpret these results to indicate that SIF1 and PentX do not assemble activated FcγR complexes at the PM. The small increase in immobile fractions of SIF1 and PentX likely reflects an increasing probability that one or more FcγR in the complex was trapped by a diffusion barrier.

We repeated these measurements using sub-activating and sub-inhibiting concentrations of Fc constructs to determine if the fractional occupancy of total FcγRs affected our SPT measurements. At low densities corresponding to ~1%–10% receptor occupancy, the tight adhesion of PMA differentiated THP-1 cells to glass appeared to result in more immobile Fc-FcγR complexes than expected. To overcome this limitation, we created SLBs displaying cyclo-RGD to allow integrin-mediated attachment. THP-1 cells were treated with the sub-activating or inhibiting concentrations (0.7 nM) multimeric Fcs at 4°C and then allowed to adhere to the cRGD-SLB at 37°C for 30 min prior to imaging by SPT-TIRF. TIRF imaging at 28 frames per second for 100 frames allowed clear resolution of low densities of DL594-Fc molecules as single particles (**Figure 4D**). As anticipated, the SLB increased the fraction of complexes undergoing free diffusion compared with cells plated on glass **(Figure 4F** vs. **Figure 4B**). Although we do not know the reasons for this, one possible explanation is that on glass, the integrin attachments may be quite static and create diffusion barriers (31), whereas on the SLB which was comprised of fluid lipids they may be free to move. Importantly, the diffusion coefficients for the rFc, SIF1 and PentX single complexes were statistically indistinguishable (**Figure 4E**), and similar to that observed on glass (**Figure 4B**), indicating that collisions with transmembrane proteins and cytosolic molecules dominated the diffusion coefficients as has been observed in apical surface SPT experiments (32, 33). Overall our diffusion coefficients measurements were nearly two times faster than those noted in literature, with the median rate of free receptors being 0.179 ± 0.012 μm^2^ per second (median ± standard error of the mean), 0.192 ± 0.011 μm^2^ per second, and 0.216 ± 0.010 μm^2^ per second, for rFc, SIF1, and PentX, compared with 0.074 ± 0.004 μm^2^/s for FcγRIIa labeled with Cy3-Fab (32) or ~0.7 μm^2^/s for FcRIIa/b & III labeled with Qdot-Fab (24). These differences may reflect the smoothness of the adherent surface of the THP-1 cells observed in TIRF here, in contrast with the ostensibly rougher non-adherent surface imaged in these other studies. Thus, FcγRs ligated with low valency Fcs retain rapid diffusion on the PM, suggesting no additional binding or aggregation of FcγR occur, even when five FcγRs are bound by PentX.

### FcγRs Move Autonomously in Preferential Sub-Domains of PM and Are Not Pre-Clustered

**Figure 5:**
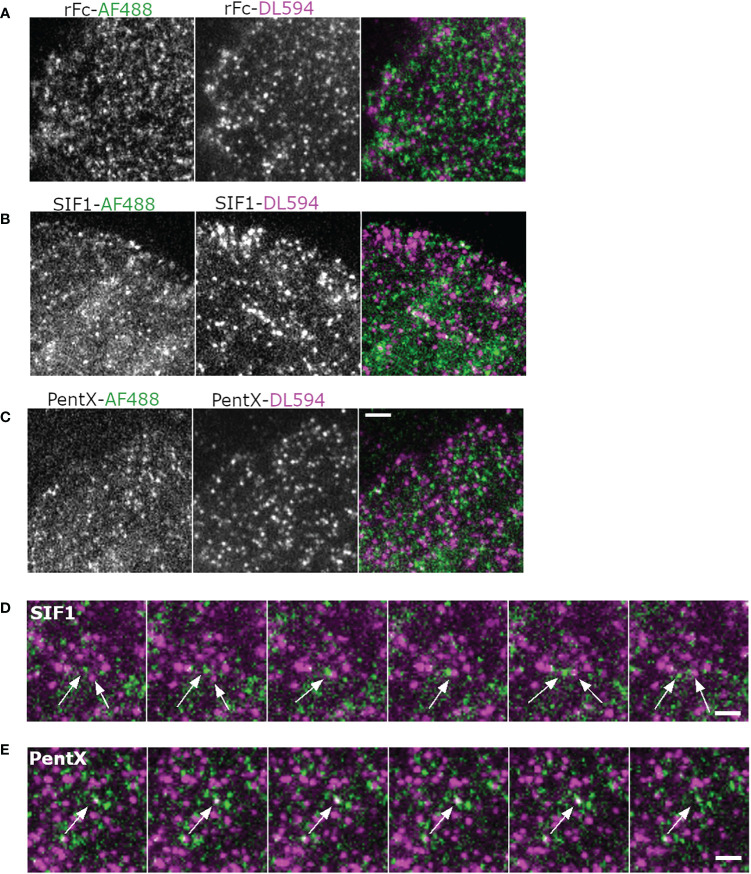
Multicolor imaging of Fc molecules indicates an absence of FcγR superclustering. **(A, B)** Montages of cells treated with AF488 and DL594-labeled rFc **(A)**, SIF1 **(B)**, PentX **(C)** at 33 µg/ml and imaged with a time-lapse of 40 s. Scale bar is 5µm. **(D)** Montage illustrating path crossing of a green and red SIF1 molecules (arrows). Scale bar is 5 µm. **(E)** Co-association and disappearance of red and green PentX, indicating a putative endocytic event. Scale bar is 5 µm.

Previous studies using super-resolution imaging in fixed cells observed pre-clustering (18) or corralling of FcγR by actin or lipid rafts and these clusters expanded when IC-IgG were added (17). To determine if FcγR pre-clustering could be observed and was remodeled by low-valency Fc binding, we imaged mixtures of the engineered Fc constructs labeled with two different fluorophores. If pre-clustering of FcγR was present, rFc of both colors should frequently be observed within the same diffraction limited sites at diminishing frequency due to the increased valency of SIF1 and PentX. Moreover, if SIF1 or PentX induce clustering on the PM beyond their binding valencies, these structures should be evident as increased frequencies of co-localized colors. The SPT data (**Figure 4**), predicts that neither preclustering nor induced clustering occur with these molecules. To test these possibilities, THP-1 cells were treated with the rFc, SIF1, and PentX, tagged with AF488 or DL594 in a 1:1 mixture at FcγR saturating concentrations (33 µg/ml of each) and imaged by two-color TIRF-360 microscopy. TIRF movies revealed that the two-color rFc molecules generally moved independently of one another and only occasionally co-localized (**Figure 5A**, **Supplementary Video 7**). Similar behavior was observed for SIF1 and PentX, with an increase in the number of confined and immobile spots, consistent with the SPT analysis (**Figures 4**, **5B, C**). Overall, Fc-multimers appeared to predominately move independently from one another despite crossing paths (**Figure 5D**, **Supplementary Video 7**). Occasionally, green and red spots could be identified within these movies, often appearing to group at immobile sites followed by both colored molecules vanishing, suggesting endocytic events (**Figure 5E**). We were unable to quantify these data using dual color SPT as the SNR for the AF488-Fc multimers was simply too low relative to the background for reliable tracking of splitting and merging events. Our qualitative analysis indicated that, rFc, SIF1, and PentX largely maintained their autonomy on the cell surface, but only occasionally would group together on the cell surface. These observations are inconsistent with extensive FcγR preclustering and contrasts sharply with clusters that form in response to IgG-docked to the SLB (**Figure 3A**, **Supplementary Video 3**) and UM (**Figure 1**), which create large cluster of FcγRs that are rapidly remodeled (16).

**Figure 6:**
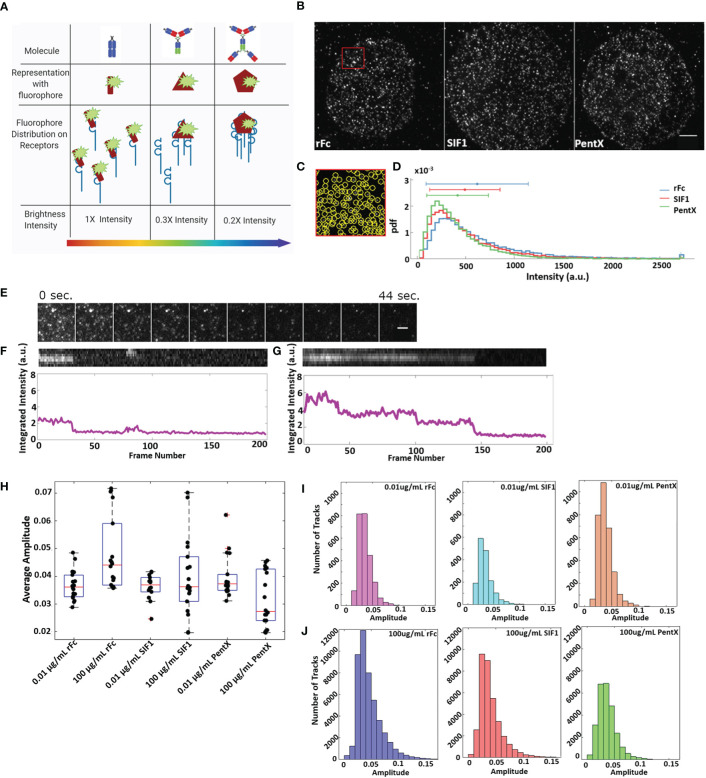
Fc receptors are not substantially preclustered and remain in small, mobile clusters upon binding multivalent Fcs. **(A)** Schematic of Fc-FcγR groupings and the resulting intensities of sub-resolution clusters. **(B)** Representative images of cells treated with 0.4 μM Fc molecule (Scale bar, 5 µm). **(C)** Zoom of the red region in B displaying individual detections of fluorescence spots. **(D)** Frequency of fluorescence of Fc molecules at 0.4 μM. Data is taken from 14 cells treated with rFc, 14 cells treated with SIF1, and 13 cells treated with PentX. **(E)** Example of stepwise photobleaching of rFc-DL594 over 200 frames taken 0.22 s apart. The rFc-DL594 was kept at 10 µg/ml to be able to track while, the rFc-AF488 was at 100 µg/ml to ensure cells remained at saturating levels. Scale bar represents 2µm. **(F)** Top: chymograph of single fluorophore bleach event from **(E)** example. Bottom: Intensity over time (frames) of bleached track. **(G)** Top: chymograph of multistep/fluorophore bleach event. Bottom: Intensity over time (frames) of the above chymograph. **(H)** Average amplitude of individual spots per cell taken over the first four frames of 100 µg/ml and 0.01 µg/ml dose multivalent Fc SPT data. **(I–J)** Histograms of single spots amplitudes averaged over 4 frames for all tracks for 0.01 µg/ml **(I)** and 100 µg/ml conditions **(J)**.

With the findings that docked Fcs moved independently **(Figure 5**, **Supplementary Video 7**) and that preferential zones of plasma membrane occupancy were evident by SPT-TIRF (**Figure 2B**), we further explored the possibility that FcγRs are grouped within microdomains of the plasma membrane. Here, we performed an intensity analysis of our SPT data to count the number of molecules within single spots, limited by the resolution of the microscope (<250nm). Fluorescent Fc molecules binding to FcγRs within very small microdomains (<50 nm), should produce single spots with brightness that scales with the number of fluorescent Fc molecules docked (**Figures 6A–D**). Histograms of all Fc-spot detections at high, equimolar, Fc concentrations on fixed cells had similar intensities, with the mean intensities of rFc>SIF1>PentX (**Figure 6D**), suggesting that Fcs could dock to microdomains containing multiple FcγRs. The differences in the means were less than a factor of two, much smaller than expected if pre-clustering was prominent amongst FcγRs. If pre-clustering of FcγRs was prevalent, the brightness of rFc spots should be maximal since multiple rFc molecules would dock within sub-resolution spots. The number of SIF1 or PentX molecules that could dock to microdomains containing a similar number of FcγRs would be reduced by ~1/3 for SIF1 and ~1/5 for PentX, resulting in a corresponding decrease in brightness. Previous work demonstrated full valency binding for these Fc multimers (6). The observed differences in intensity were much smaller, ~20% decrease from rFc to SIF1 and ~35% decrease from rFc to PentX. We conclude that the similar intensity histograms represent FcγRs generally dispersed over a larger ~ 50 nm corrals. This finding is consistent with the SPT tracks spanning hundreds of nanometers within 200 s (**Figures 2C**, **3B**), and thus, a chance of coincident occupation of FcγRs within a given microdomain is transient, consistent with the observations of the 2-color experiments (**Figure 5**). Thus, our data supports a model where corrals on the cell surface weakly constrain FcγR movements, but they do not trap them in pre-formed clusters in when bound with rFc, SIF1, or PentX.

Detailed analysis of fluorescent intensities from SPT data revealed that many tracked spots exhibited single stepwise photobleaching indicating a single fluorophore (**Figures 6E, F**). Occasionally multiple photobleaching steps indicative of multiple fluorophores could be observed (**Figure 6G**) indicating multiple Fcs or multiple dyes per Fc molecule. At low concentrations of Fc molecules (0.01 µg/ml) the spot intensities were indistinguishable across rFc, SIF1, and PentX (**Figure 6I**) indicating that they predominantly reflected single Fc molecules docked to FcγRs, supporting the notion that low valency Fcs did not create larger FcγR clusters. Moreover, at saturating doses (100 μg/ml) of rFc, SIF1 or PentX, the preponderance of spots in live cells displayed similar spot-intensity histograms (**Figure 6J**). For PentX, the histograms were nearly identical between low and saturating concentrations. For SIF1, and to a greater degree, rFc, the histograms displayed tails to the right, indicating that coincident detections were more common owing to more frequent labeling of multiple FcγR within a sub-resolution spot. Taken together, these results are consistent with a model in which FcγRs move independently within large corrals with some that are weakly trapped or immobilized in regions of the plasma membrane, yet changing their oligomerization states when docked with rFc, SIF1, or PentX had no impact on this trapping. Likely only activating valencies of Fcs such as encountered in IgG-IC (**Figure 1**) or surface associated IgG **(Figure 3A**) are able to remodel FcγR distributions on the cell surface.

## Discussion

**Figure 7:**
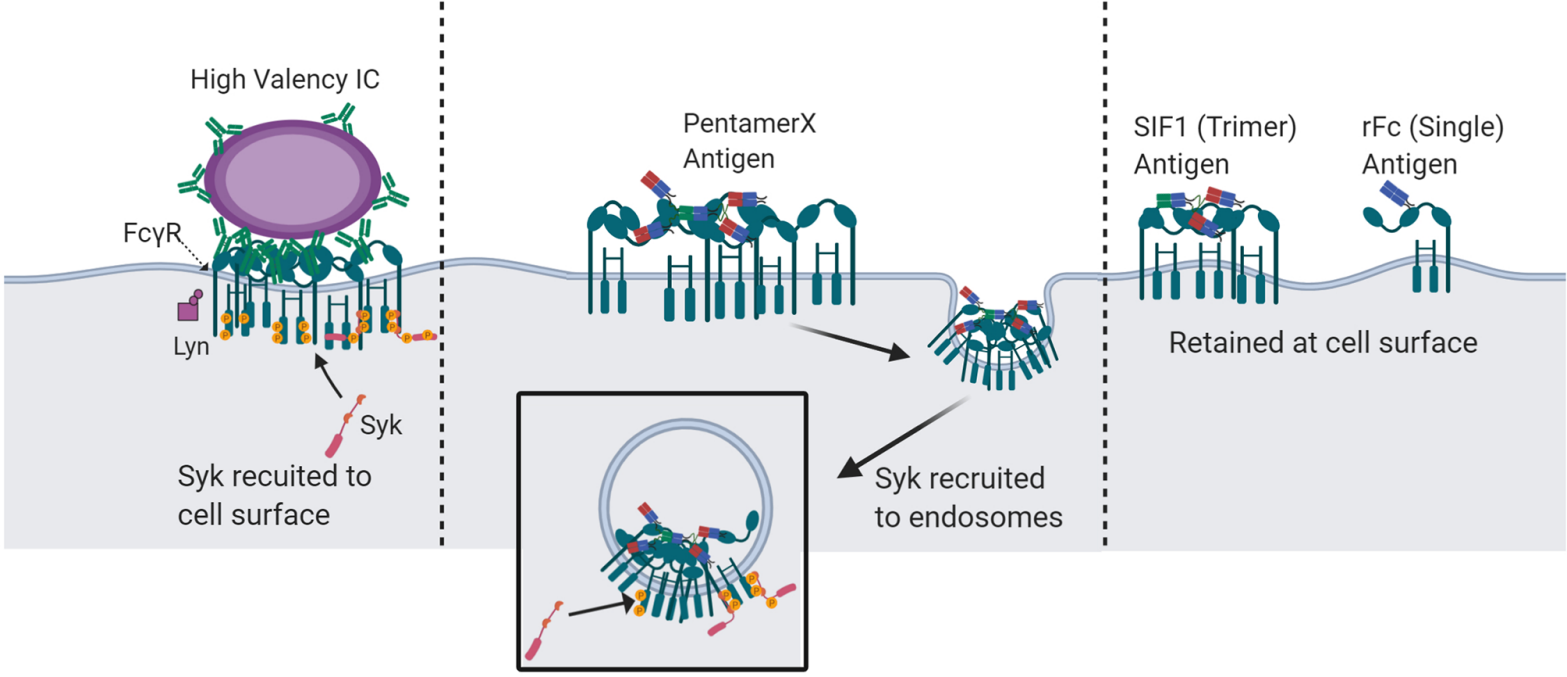
Proposed mechanisms of spatio-temporal FcγR activation and Syk recruitment by multivalent-Fc molecules. IC-IgG : FcγR clusters at the plasma membrane with a valency of more than five Fc domains undergo Syk recruitment and activation at the cells surface. IC : FcγR clusters with a valency of five or fewer do not robustly recruit Syk to the plasma membrane and are unable to fully activate at the cell surface. However, at the valency of five, in a pentameric geometry, FcγR clusters are endocytosed and recruit Syk at the endosome. Molecules with less than 5 Fc domains can engage FcγRs at the cell surface but cannot recruit Syk. Created with BioRender.com.

The engineered multimeric Fc molecules, rFc, SIF1, and PentX, provided new insight into the minimal number of FcγRs that must be engaged for signaling and that this threshold depends on the subcellular environment. While the classical models for FcγR activation predict a minimal number of ligated and clustered receptors is necessary for activation, our findings give the surprising result that endosomes may afford a lower threshold of activation for the minimum number of FcγRs than the plasma membrane. Specifically, we found that PentX, which potently drives Syk phosphorylation through the binding of five FcγRs (6), can only fully activate FcγRs within endosomes (**Figure 7**). This result could be explained by two potential mechanisms. First, endosomes may allow segregation of the FcγRs from plasma membrane associated phosphatases to allow full activation. Alternatively, sorting of PentX-FcγRs within endosome membrane may result in compartmental or sub-compartmental sorting that creates elevated concentrations of PentX-FcγRs enabling FcγR activation. A notable feature of these results was that PentX accumulated and was trafficked to large endosomes whereas SIF1 and rFc were only evident on small endosomes at similar times of incubation (**Figure 2**). Additionally, we found that Syk was required for the accumulation of PentX within large endosomes, and its absence resulted in PentX traffic that was similar to rFc and SIF1 (**Figure 3**). These observations support a model in which Syk recruitment to endosomal FcγRs may facilitate compartmental or sub-compartmental sorting by trapping PentX-FcγRs. Conversely, SIF1 and rFc do not recruit Syk to endosomes displayed slower internalization and endosomal traffic, which was faster in the likely more endocytic FLM than THP-1 cells. Another possibility, supported by the observation of vesicles bearing SIF1 and rFc but were dim and they did not recruit Syk, is that they are transient intermediate of membrane recycling. An implication of this finding is that IC-IgGs could preferentially activate FcγRs on endosomes, where FcγR signals may be unique from those generated at the PM. The PentX molecule provides a powerful new tool for studying the properties of endosomal FcγR-specific signaling that cannot be accessed as precisely with heterogenous IC-IgG preparations.

Our finding, that FcγRs on the PM are dynamically confined, but not preclustered on the PM, and that their threshold for activation requires more than five FcγRs sets a lower bound on the minimum number of FcγRs needed to form signaling competent clusters on the PM. While exactly how FcγR rearrangements generate productive signaling, our results are consistent with the actin cytoskeleton and potentially other transmembrane proteins acting as fences corralling FcγRs (29, 33, 34). These interactions are likely important for organizing FcγRs activation during engagement of particle associated IgG by sorting activated FcγRs into regions that have close spacing between the phagocyte membrane and target, allowing exclusion of bulky and glycosylated phosphatases such as CD45 (29, 35). More work will be needed to determine if initiation of FcγR phosphorylation by Src-family kinases, including Lyn, occurs on FcγRs docked with PentX or similar low valency molecules, but is insufficient to recruit Syk or is rapidly reversed at PM. Our finding here that large Fc oligomers (UM) were able to self-organize into patches at the PM suggests that this sorting process does not require an opposing target membrane for clustering FcγRs into PM patches, however it is possible that CD45 exclusion could occur by the molecular crowding of many Fc/FcγRs complexes. Moreover, super resolution imaging indicated that of IC-IgGs made up of 2,4-dinitrophenyl-bovine serum albumin bearing approximately 12-24 anti-DNP IgGs were sufficient to promote clustering and activation on human monocytes (17), implying that the number of ligated FcγRs required for signaling is between 6 and 12. In contrast to the work by Brandsma et al., in which pre-patching of FcγRs that were expanded by IC-IgG observed in fixed cells, we did not find evidence of pre-formed FcγR clusters or altered FcγR distributions for low-valency Fc molecules. Rather, our SPT traces in living cells showed zones of preferential dynamic occupancy and exclusion typically containing tracks from free and confined receptors with D ~ 0.15–0.2 μm^2^/s suggesting that rather than pre-formed clusters, the FcγRs are corralled by actin (29). Indeed, observed confined diffusion trajectories had dimensions similar to the cluster diameters (~ 50 nm) observed by Brandsma et al. (17). This distinction has important consequences for IC induced signaling and FcγR activation and inhibition, as both the number of ligated FcγRs and their spatial organization across membrane microdomains appears to be a critical for controlling their activation.

The mechanism of SIF1 inhibition of FcγR signaling elucidated here, indicates that FcγRs can be inhibited without activation or promoting degradation beyond membrane turnover. At the plasma membrane, SIF1-FcγR complexes had nearly indistinguishable motions as rFc-FcγR and none recruited Syk-mScarlet, indicating that SIF1 occupied the IgG binding sites while trapping the FcγR in a non-activatable state. Within endosomes, SIF1 and rFc docked FcγRs were few and transient indicating that they lingered on the PM, with a relatively slow internalization kinetic. We interpret these findings as SIF1 binds FcγRs but does not alter their native inactive state in any subcellular compartment, even with the lower activation thresholds as implied by the PentX results. SIF1 is an attractive inhibitor as it has significantly higher overall affinity than rFc or IVIG, arising from avidity by binding three FcγRs, leading to therapeutic effects at lower drug concentrations against immune complex mediated diseases (6). Moreover, SIF1 can likely engage with all FcγR types while maintaining the properties of a single receptor making it ideal to displace pathological IC-IgGs or to disrupt particle opsonized IgG responses such as antibody dependent cellular cytotoxicity or phagocytosis.

In contrast to conventional thinking, internalization from plasma membrane in this system does not appear to be a result of Syk recruitment to ligated FcγRs, but rather provides a microenvironment for FcγR signaling and Syk recruitment in subcellular membranes. A remaining question is do the multivalent Fcs promote Syk recruitment in endosomes or is internalization of the Fc/FcγR complexes a default pathway that through removal of the complex from the PM, facilitates FcγR activation? Our data suggest that in the case of SIF1 and rFc, no detectable Syk recruitment is observed on internal membranes or the PM, despite endocytic traffic of SIF1 and rFc. Furthermore, the long residence time of SIF1 and rFc on the cell surface, suggests that these molecules docked to FcγRs are dynamically being internalized and recycled back to the cell surface.

## Data Availability Statement

The original contributions presented in the study are included in the article/**Supplementary Material**. Further inquiries can be directed to the corresponding author.

## Ethics Statement

The animal study was reviewed and approved by South Dakota State University Institutional Animal Care and Use Committee.

## Author Contributions

AC, CB, EB, and AH designed the experimental plan and evaluated data. EB, DO, JS, AM, and AH conducted the experiments and analyzed the data. EB, AC, CB, and AH wrote the paper. All authors contributed to the article and approved the submitted version.

## Funding

This work was supported by funds provided by Momenta Pharmaceuticals as well as the NSF/EPSCoR Cooperative Agreement #IIA-1355423, the South Dakota Research and Innovation Center, BioSNTR, and by the State of South Dakota BOR CRGP to AH. EB was supported through the ASERT-IRACDA program (K12GM088021).

## Conflict of Interest

AC, DO, JS, AM, and CB were employed by Momenta Pharmaceuticals during the project and have equity interest. CB, AM, and DO are inventors on patent application PCT/US2015/028926 (WO/2015/168643) submitted by Momenta Pharmaceuticals that covers “Compositions and methods related to engineered Fc constructs”.

The remaining authors declare that the research was conducted in the absence of any commercial or financial relationships that could be construed as a potential conflict of interest.

## Supplementary Material

Supplementary Video 1 Syk-mScarlet redistribution to patches of Uncontrolled Multimer (UM) AF488 (100 µg/ml). The confocal movies from left to right include the UM AF488, Syk-mScarlet, and the overlay of the two, with FcM pseudo-colored green and the Syk-mScarlet pseudo-colored magenta. Movies are 50 frames with time between frames being 0.292 s. Scale Bar represents 5μm.

upplementary Video 2 Redistribution Syk-mScarlet, to internalized PentX-AF488 (100 µg/ml). The confocal movies from left to right include the PentX AF488, Syk-mScarlet, and the overlay of the two, with PentX pseudo-colored green and the Syk-mScarlet pseudo-colored magenta. Movies are 50 frames at 0.292 s between frames. Scale Bar represents 5μm.

Supplementary Video 3 Syk-mScarlet is recruited to patches of FcγRs moving on an IgG opsonized bilayer. TIRF imaging show THP-1 cells engaging an IgG-AF647 opsonized bilayer, driving FcγR clustering, and Syk-mScarlet recruitment to clustering receptors. In the merge, green represents IgG-AF647 and the magenta represents Syk-mScarlet. 100 frames at 1 s between frames. Scale Bar represents 5μm.

upplementary Video 4 TIRF imaging of Syk-mScarlet in THP-1 cells treated with 100 µg/ml rFc-AF488. In the merged movie, rFc is pseudo-colored green and Syk-mScarlet is pseudo-colored magenta. The movies are 100 frames with 0.120 s between frames. Scale bar represents 5μm.

Supplementary Video 5 TIRF imaging of Syk-mScarlet in THP-1 cells treated with 100 µg/ml SIF1-AF488. In the merged movie, SIF1 is pseudo-colored green and Syk-mScarlet is pseudo-colored magenta. The movies are 100 frames with 0.120 s between frames. Scale bar represents 5μm.

Supplementary Video 6 TIRF imaging of Syk-mScarlet in THP-1 cells treated with 100 µg/ml PentX-AF488. In the merged movie, PentX is pseudo-colored green and Syk is pseudo-colored magenta. The movies are 100 frames with 0.120 s between frames. Scale bar represents 5μm.

Supplementary Video 7 THP-1 cells with a 1:1 mixtures of Fc multimers labeled with two different fluorophores. The molecules, rFc, SIF1, and PentX, were conjugated with either AF488 or DyL594 to investigate super clustering. The left panel shows THP-1 cells with docked rFc-AF88, pseudo-colored green, and rFc-DL594, pseudo-colored magenta. The middle panel shows THP-1 cells with docked SIF1-AF88, pseudo-colored green, and SIF1-DL594, pseudo-colored magenta. The right panel shows THP-1 cells with docked PentX-AF88, pseudo-colored green, and PentX-DL594, pseudo-colored magenta. The two-color movies are 50 frames with 0.220 s between frames, concentrations were 33 µg/ml for AF488- Fcs and 33µg/ml AF594-Fcs. The scale bar represents 5μm.
