# Supplementary material for: Engineered IgG1-Fc Molecules Define Valency Control of Cell Surface Fcγ Receptor Inhibition and Activation in Endosomes
Source: Front Immunol. 2021 Feb 15;11:617767. doi: 10.3389/fimmu.2020.617767 (PMC7928370; doi:10.3389/fimmu.2020.617767)
Supplement: Supplementary file 8 [file DataSheet_1.pdf]

Supplementary Material

Supplementary Figures

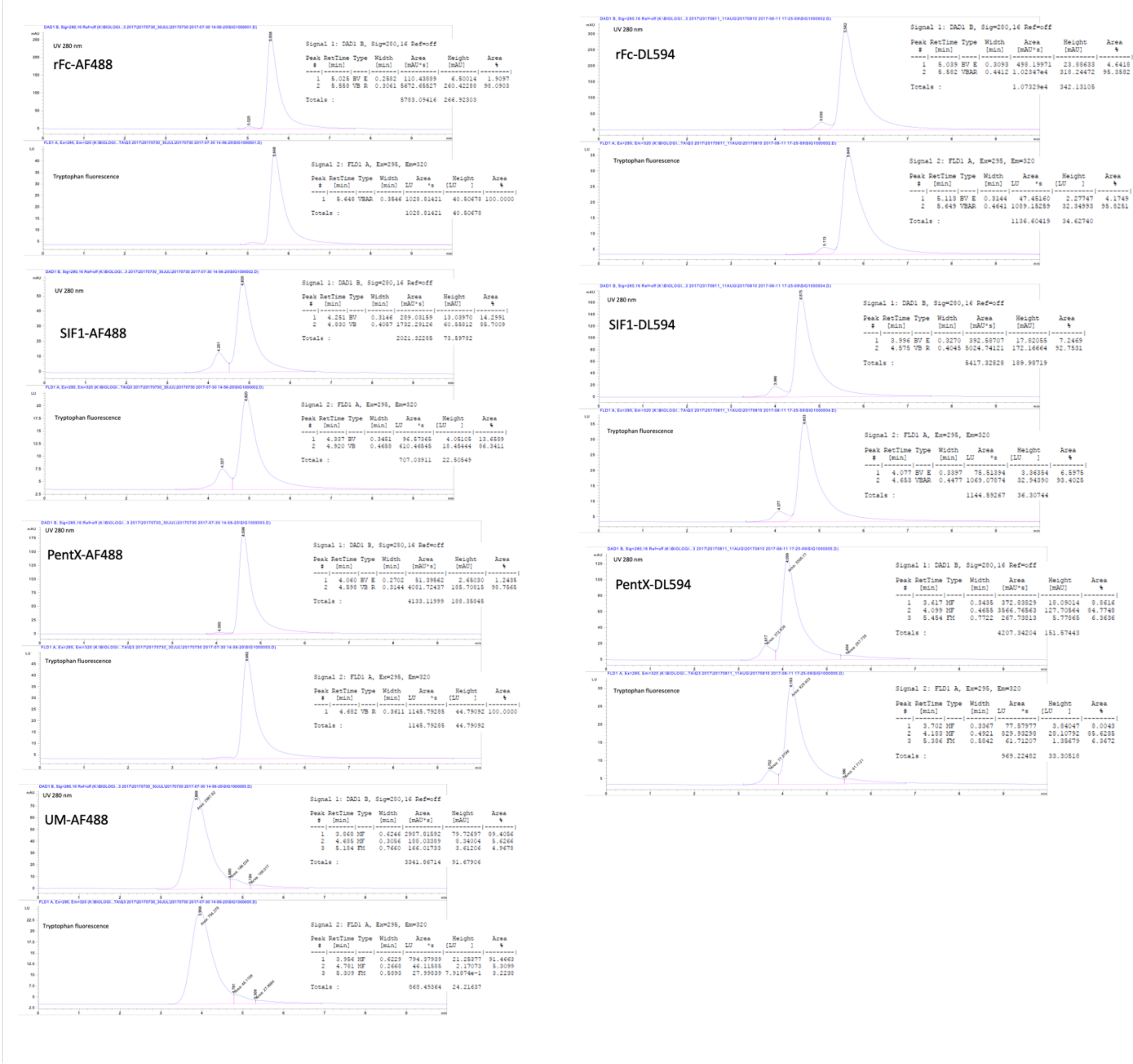

Supplementary Figure 1. Size exclusion chromatography confirming the purity and size distributions of mutimeric Fc molecules used. Fluorescently-labeled molecules as indicated, were analyzed by size exclusion chromatography on an Agilent 1200 series HPLC equipped with UV and fluorescence detectors. Used a Xenix SEC-300, 4.6x300 mm, 3 um, 300 Å column (Sepax Technologies) with an isocratic 150 mM sodium phosphate pH 7.0 mobile phase.

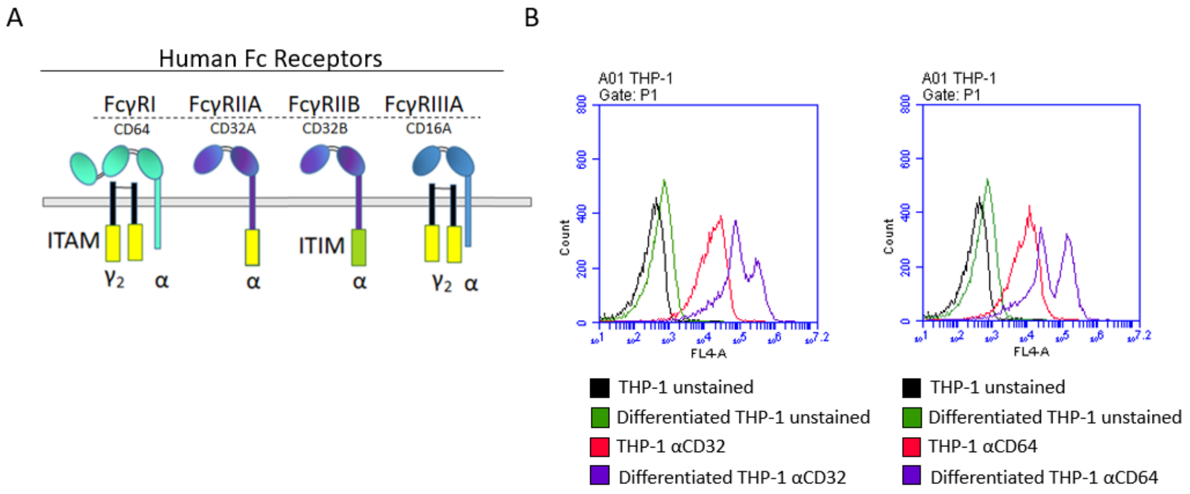

Supplementary Figure 2. PMA differentiation increases expression of Fcγ Receptors. (A) Human FcγRs. (B) Cell surface abundance of of CD32 (left) and CD64 (right) measured by anti-CD32 and anti-CD64 fab fragments after before and after differentiation with Phorbol 12-myristate 13-acetate (PMA).

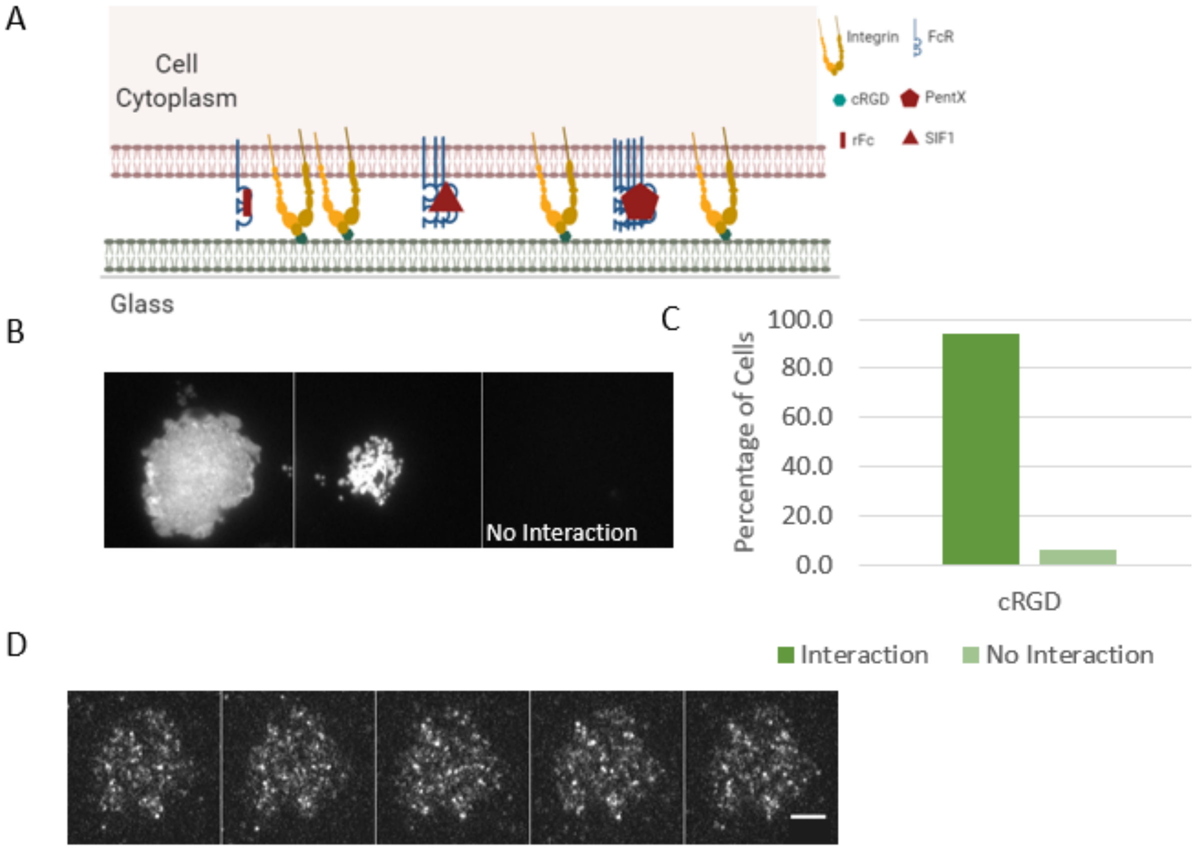

Supplementary Figure 3. cRGD-functionalized Supported Lipid Bilayer (SLB) supports macrophage attachment. A) Illustration of integrins engaged with cRGD-SLB and Fc-FcγR complexes (created with BioRender). (B) TIRF imaging of THP1 cells labeled with DiI attached to cRGD-SLB. Cells that do not bind are visible in Epi but not in TIRF field when they do not interact with the cRGD-SLB. (C) Quantified interaction of DiI labeled interacting with cRGD-SLB. (D) Example of rFc DL594 distributions on THP-1 cells interacting with cRGD-SLB.
